# Visualization of three-way comparisons of omics data

**DOI:** 10.1186/1471-2105-8-72

**Published:** 2007-03-05

**Authors:** Richard Baran, Martin Robert, Makoto Suematsu, Tomoyoshi Soga, Masaru Tomita

**Affiliations:** 1 Institute for Advanced Biosciences, Keio University, Tsuruoka, Yamagata 997-0017, Japan; 2 Department of Biochemistry and Integrative Medical Biology, School of Medicine, Keio University, Shinanomachi, Shinjuku-ku, Tokyo 160-8582, Japan; 3 Present address: Institute of Chemistry, Slovak Academy of Sciences, Dúbravská cesta 9, 845 38 Bratislava, Slovakia

## Abstract

**Background:**

Density plot visualizations (also referred to as heat maps or color maps) are widely used in different fields including large-scale omics studies in biological sciences. However, the current color-codings limit the visualizations to single datasets or pairwise comparisons.

**Results:**

We propose a color-coding approach for the representation of three-way comparisons. The approach is based on the HSB (hue, saturation, brightness) color model. The three compared values are assigned specific hue values from the circular hue range (e.g. red, green, and blue). The hue value representing the three-way comparison is calculated according to the distribution of three compared values. If two of the values are identical and one is different, the resulting hue is set to the characteristic hue of the differing value. If all three compared values are different, the resulting hue is selected from a color gradient running between the hues of the two most distant values (as measured by the absolute value of their difference) according to the relative position of the third value between the two. The saturation of the color representing the three-way comparison reflects the amplitude (or extent) of the numerical difference between the two most distant values according to a scale of interest. The brightness is set to a maximum value by default but can be used to encode additional information about the three-way comparison.

**Conclusion:**

We propose a novel color-coding approach for intuitive visualization of three-way comparisons of omics data.

## Background

Color-coded representations of differences between omics datasets provide an intuitive and global comparative view of the data [1]. Such visualizations further facilitate the use of human pattern recognition abilities to complement the automated approaches to pinpoint subtle differences [2]. Currently, most visualizations are limited to pairwise comparisons where differences of interest between two corresponding datapoints are mapped onto color gradients for positive or negative ranges. In addition, results of statistical tests (F ratio, *z*-score, quartile analysis, etc.) performed across multiple datasets can be visualized to highlight sets of corresponding datapoints containing a difference [3]. These results, however, do not provide information about the actual distribution of the corresponding datapoints – which of them are similar or different. Often, three (sets of) omics datasets are compared to gain insight into biological function [4-8]. Intuitive three-way comparisons can further be useful for specific applications such as in drug discovery where therapeutic equivalence studies may include a control and two different treatments, namely the tested and accepted drug and a new compound under development.

Here, we propose a novel color-coding approach for the visualization of three-way comparisons. The approach is based on the HSB (hue, saturation, brightness) color model [9]. The hue component of the HSB color model provides a convenient way to perform smooth color transitions making it a popular choice for density plot (color map, heat map) visualizations. We also employ another feature of the hue component, namely its circular nature, to perform mappings of possible distributions of three compared values onto the color space. The proposed color-coding approach facilitates intuitive overall visualization of three-way comparisons of large datasets.

## Results

**Figure 1:**
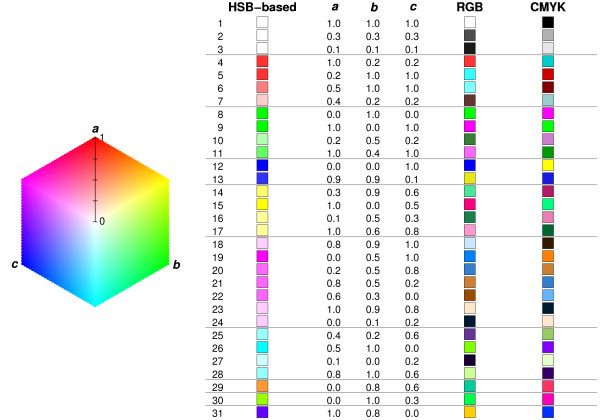
**Examples of color-codings for three-way comparisons**. Color representations for three-way comparisons of selected values *a*, *b*, and *c *calculated using the proposed procedure are shown in the column labeled HSB-based. Colors acquired by substituting values of *a*, *b*, and *c *directly for red, green, and blue or cyan, magenta, and yellow (black = 0) are shown in columns labeled RGB or CMYK, respectively. The legend is drawn as a hexagon instead of a circle for convenience. Horizontal lines separate groups of values with similar distributions.

The basic color scheme, based on the HSB model, is shown in Figure 1 together with color representations for three-way comparisons of selected sets of values. The color representations were calculated according to the proposed procedure described in the Methods section. When the three compared values are identical, the resulting color is white (Figure 1, rows 1–3). If two of the values are identical and one of them is different, the resulting color corresponds to the hue characteristic of the differing value. For example, if *a *is the different value, the resulting color is red (rows 4–7); if *b *is the different value, the resulting color is green (rows 8–11); and if *c *is the different value, the resulting color is blue (rows 12 and 13).

When all three values to be compared are different, the color representing their three-way comparison is selected from the color gradient running between the characteristic hues of the two most distant values (measured by the absolute value of their difference). The exact color depends on the relative position of the remaining value between the two most distant values. If *a *and *b *are the most distant values and *c *lies half way between them, the resulting color is yellow (rows 14–17). If *c *lies closer to *b*, the color becomes orange (row 29) and if *c *lies closer to *a*, the color becomes yellow-green (row 30). Similarly, if *a *and *c *are the most distant and *b *lies half-way between them, the resulting color is pink (rows 18–24). If *b *and *c *are the most distant and *a *lies half-way between them, the resulting color is cyan (rows 25–28).

The saturation of the colors indicates the extent of differences between the values. When two of the compared values are identical and one is different, the saturation value corresponds to the distance between the two identical values and the unique value (e.g. rows 4–13). If all three values are different, the saturation corresponds to the distance between the two most distant values (e.g. rows 18–28).

To contrast other color schemes with our proposed color-coding method, Figure 1 also shows colors which result from direct substitutions of the compared values into RGB (red, green, blue) and CMYK (cyan, magenta, yellow, black) color models. Identical values lead to colors from white to black (grayscale) gradient for both color models (rows 1–3). Distributions, in which two compared values are identical and one is different (rows 4–13) can each be represented by one of two colors with varying brightness. If *a *≠ *b *= *c*, direct RGB coding leads to red if *a *> *b *= *c *(rows 4 and 7) or cyan if *a *<*b *= *c *(rows 5 and 6). For both RGB and CMYK direct coding, using two colors per distribution group (separated by horizontal lines in Figure 1) may provide additional distinguishing features for individual distributions, but also lead to undesirable ambiguities. For example, the RGB colors for rows 18–20 corresponding to *a *≠ *b *≠ *c *and *b *lies half-way between *a *and *c *are very similar to cyan, corresponding to *a *≠ *b *= *c *(rows 5,6) and blue corresponding to *a *= *b *≠ *c *(row 12). Other similar sources of ambiguity can be found in both RGB and CMYK columns of Figure 1. Moreover, the brightness of the colors given by direct RGB or CMYK coding cannot be interpreted easily. For RGB direct coding, in some cases smaller absolute differences lead to darker colors (e.g. rows 4 and 7) while in other cases identical absolute differences lead to different brightness of the color (rows 21 and 22). For all these reasons the proposed color-coding approach appears superior for intuitive visualization of three-way comparisons. 

**Figure 2:**
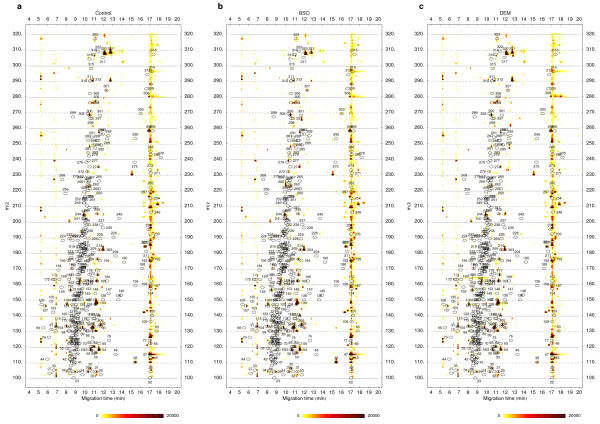
**Metabolite profiles for the three-way comparison**. Mouse liver extract metabolite profiles acquired by CE-TOFMS two hours after intraperitoneal injection with (**a**) vehicle (Control), (**b**) diethylmaleate (DEM), a non-protein thiol-depleting chemical, or (**c**) buthionine sulfoximine (BSO), an inhibitor of γ-glutamylcysteine synthase. The plotted datasets are averages of five normalized replicate datasets for cation measurements originating from our previous work [2]. The averaged datasets are visualized as density plots. For all plots, numbered ovals (annotation labels) indicate the expected locations of peaks of a set of known chemical compounds and are used for identification of metabolites on the density plots [2,3].

**Figure 3:**
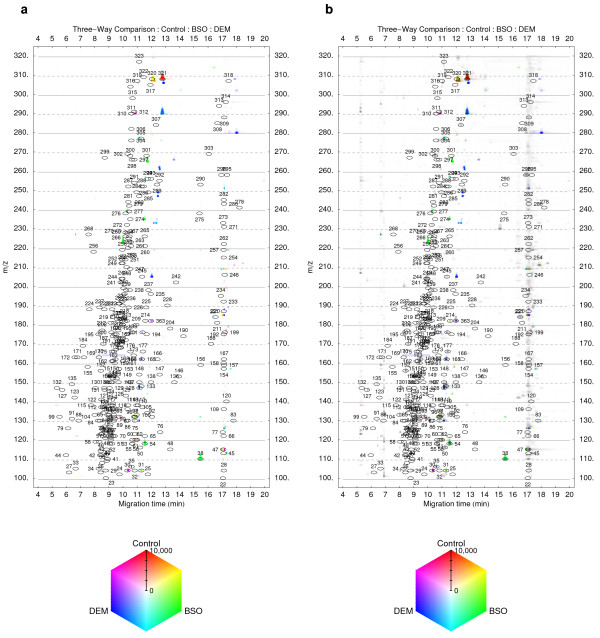
**Three-way comparison of metabolite profiles**. (**a**) Absolute × relative three-way comparison of metabolite profiles shown in Figure 2. Averages of replicate datasets (n = 5) were used for the three-way comparison. The resulting dataset was filtered using F-ratio (one-way ANOVA) to select only statistically significant differences as described in the main text. (**b**) The Control dataset (Figure 2a) was overlaid on the three-way comparison result shown in panel (a) via the brightness value. Darkening of the colored spots indicates the size of the corresponding peaks in the Control dataset. Gray spots show peaks which do not significantly differ among the datasets. For both plots, numbered ovals (annotation labels) indicate the expected locations of peaks of a set of known chemical compounds and are used for identification of metabolites on the density plots [2,3].

To illustrate how the visualization method can be used to analyze experimental data, we applied the proposed color-coding method to direct three-way comparisons of metabolite profiles. Three groups of replicate quantitative metabolite profiles (n = 5) derived from capillary electrophoresis time-of-flight mass spectrometry (CE-TOFMS) analysis of mouse liver samples were used for the comparison. The datasets originate from our previous work [2]. Replicate datasets from each group were normalized and averaged into single datasets which are visualized as density plots in Figure 2. In this case the data is represented in three dimensions as a map of signals in time (x-axis), molecular mass (m/z), and intensity (color). An additional filter dataset was generated by calculating the F ratio (one-way ANOVA) for the groups of all corresponding signal intensities from the original replicate datasets. A moving average smoothing filter (window size 9) was applied to all electropherograms in the filter dataset. The averaged datasets (Figure 2) were used for the generation of an initial three-way comparison result (not shown). This preliminary comparison was then processed to remove signals for which the corresponding F ratio value in the filter dataset was below a threshold value of 3.9 (corresponding to *p *= 0.05 when comparing three groups of five replicate values). The final filtered three-way comparison result is shown if Figure 3a.

**Figure 4:**
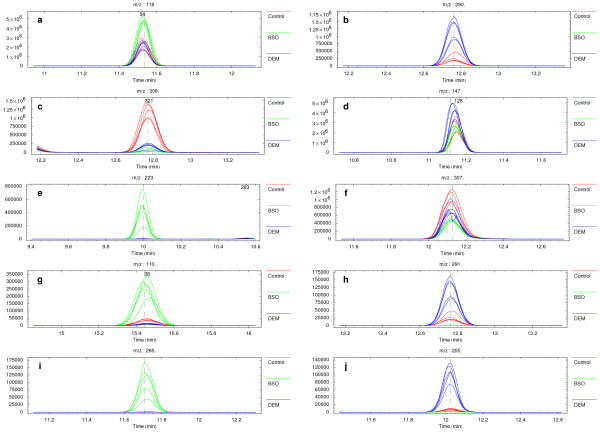
**Candidate differences**. Overlaid extracted ion electropherograms for the most significant differences from the three-way comparison results shown in Figure 3. Each panel represents data in the form of signal intensity (number of ions) over time for a specific mass interval (1 Da bin). The vertical dashed line indicates the position of the most significant difference according to the three-way comparison results. When present within panels, numbers correspond to the annotation labels in Figures 2 and 3.

Parts of the data corresponding to the vicinity of the most significant differences according to the three-way comparison results (Figure 3a) in the normalized replicate datasets are shown in Figure 4 in the form of overlaid extracted electropherograms. These represent the mass electropherograms of metabolite profiles obtained from CE-TOFMS and are used here to confirm visually that the signals are genuine and not due to noise or other artifacts.

Multiple types of possible distributions of compared values, as discussed above, are visible in Figure 3a. Distributions in which one specific value is different and the remaining two compared values are similar are shown as red (label 321 in Figures 2 and 3, corresponding to Figure 4c), green (labels 54 and 38, Figure 4a, g), or blue (near label 245, Figure 4j). Distributions in which all three of the compared values are different and one value lies approximately half-way between the remaining two are shown as yellow (near label 320, Figure 4f), pink (near label 312), or cyan (near label 305).

As described in the Methods section, the brightness value of the HSB color model is not used in the proposed color-coding method but can be used to encode additional information about the three-way comparison. For example, Figure 3b shows an overlay of one of the compared averaged datasets (Figure 2a) onto the filtered three-way comparison result (Figure 3a) via the brightness value. This results in a darkening in the color of the spots that is proportional to the size of the corresponding peaks in the overlaid dataset. Peaks, which do not differ significantly among the three compared averaged datasets, lead to no signals on the filtered three-way comparison result (Figure 3a), but appear as gray spots in Figure 3b (e.g. labels 50, 177, 300) providing both a global overview of total sample composition and instant visualization of specific differences.

## Discussion

Visualizations using the proposed color-coding approach provide intuitive overall views for three-way comparisons of large datasets. These visualizations further allow identification of signals different specifically in one of the three datasets or signals different for all three compared datasets.

One limitation of the proposed color-coding is that distributions such as *a *> *b *= *c *and *a *<*b *= *c *produce the same result. In other words, if red, green, and blue are the characteristic hues for the three compared values, a red coloration only indicates that *b *and *c *are identical and that *a *is different. It does not specify whether *a *is greater than or smaller than the other two. Similarly, a yellow coloration indicates that *a *and *b *are the most distant values while *c *lies half-way between them. It does not specify which of *a *or *b *is greater than *c*. However, in most cases, simply knowing which of the three values are similar or different is the main objective and may be sufficient initially. The exact distribution can be confirmed subsequently (e.g. on the chromatograms generated for candidate differences) or undesirable distributions can be filtered out from the three-way comparison results.

Alternative color-coding approaches for three-way comparisons are also possible. For example, normalizing the three compared values and using these directly as specifiers for the RGB (red, green, blue) color model provides a unique color-coding. However, the resulting colors do not represent the three-way differences as intuitively as the colors generated by the proposed approach.

## Conclusion

The proposed color-coding approach allows intuitive overall visualizations of three-way comparisons of large datasets. The approach was demonstrated with metabolomic datasets but it can equally be applied to extend the visualizations of pairwise comparisons of gene expression data [1,10,11] or pathway-based visualizations [12,13] to three-way comparisons. Beyond omics data visualization in biological research, the generic nature of the color-coding approach is likely to extend its applicability to an even wider array of data analysis fields where a visual comparison of any three signals is desirable.

## Methods

### Color-coding for three-way comparisons

The color-coding for the representation of a three-way difference between three corresponding datapoints (*a*, *b*, and *c*) is based on the HSB (hue, saturation, brightness; ranges from 0 to 1) color model. The hue value of the color representing the three-way comparison of *a*, *b*, and *c *is calculated using one of the following equations, according to the signals distribution:

Hresult={0∗fora=b=cHa+(Hb−Ha)|b−c||a−b|for|a−b|≥|b−c|∧|a−b|≥|a−c|Hb+(Hc−Hb)|a−c||b−c|for|b−c|>|a−b|∧|b−c|≥|a−c|Mod[Hc+(Ha+1−Hc)|a−b||a−c|,1]for|a−c|>|a−b|∧|a−c|>|b−c|
 MathType@MTEF@5@5@+=feaafiart1ev1aaatCvAUfKttLearuWrP9MDH5MBPbIqV92AaeXatLxBI9gBaebbnrfifHhDYfgasaacH8akY=wiFfYdH8Gipec8Eeeu0xXdbba9frFj0=OqFfea0dXdd9vqai=hGuQ8kuc9pgc9s8qqaq=dirpe0xb9q8qiLsFr0=vr0=vr0dc8meaabaqaciaacaGaaeqabaqabeGadaaakeaacqWGibasdaWgaaWcbaGaemOCaiNaemyzauMaem4CamNaemyDauNaemiBaWMaemiDaqhabeaakiabg2da9maaceqabaqbaeqabqWaaaaabaGaeGimaaZaaWbaaSqabeaacqGHxiIkaaaakeaacqqGMbGzcqqGVbWBcqqGYbGCaeaacqWGHbqycqGH9aqpcqWGIbGycqGH9aqpcqWGJbWyaeaacqWGibasdaWgaaWcbaGaemyyaegabeaakiabgUcaRiabcIcaOiabdIeainaaBaaaleaacqWGIbGyaeqaaOGaeyOeI0IaemisaG0aaSbaaSqaaiabdggaHbqabaGccqGGPaqkdaWcaaqaamaaemaabaGaemOyaiMaeyOeI0Iaem4yamgacaGLhWUaayjcSdaabaWaaqWaaeaacqWGHbqycqGHsislcqWGIbGyaiaawEa7caGLiWoaaaaabaGaeeOzayMaee4Ba8MaeeOCaihabaWaaqWaaeaacqWGHbqycqGHsislcqWGIbGyaiaawEa7caGLiWoacqGHLjYSdaabdaqaaiabdkgaIjabgkHiTiabdogaJbGaay5bSlaawIa7aiabgEIizpaaemaabaGaemyyaeMaeyOeI0IaemOyaigacaGLhWUaayjcSdGaeyyzIm7aaqWaaeaacqWGHbqycqGHsislcqWGJbWyaiaawEa7caGLiWoaaeaacqWGibasdaWgaaWcbaGaemOyaigabeaakiabgUcaRiabcIcaOiabdIeainaaBaaaleaacqWGJbWyaeqaaOGaeyOeI0IaemisaG0aaSbaaSqaaiabdkgaIbqabaGccqGGPaqkdaWcaaqaamaaemaabaGaemyyaeMaeyOeI0Iaem4yamgacaGLhWUaayjcSdaabaWaaqWaaeaacqWGIbGycqGHsislcqWGJbWyaiaawEa7caGLiWoaaaaabaGaeeOzayMaee4Ba8MaeeOCaihabaWaaqWaaeaacqWGIbGycqGHsislcqWGJbWyaiaawEa7caGLiWoacqGH+aGpdaabdaqaaiabdggaHjabgkHiTiabdkgaIbGaay5bSlaawIa7aiabgEIizpaaemaabaGaemOyaiMaeyOeI0Iaem4yamgacaGLhWUaayjcSdGaeyyzIm7aaqWaaeaacqWGHbqycqGHsislcqWGJbWyaiaawEa7caGLiWoaaeaacqqGnbqtcqqGVbWBcqqGKbazdaWadaqaaiabdIeainaaBaaaleaacqWGJbWyaeqaaOGaey4kaSIaeiikaGIaemisaG0aaSbaaSqaaiabdggaHbqabaGccqGHRaWkcqaIXaqmcqGHsislcqWGibasdaWgaaWcbaGaem4yamgabeaakiabcMcaPmaalaaabaWaaqWaaeaacqWGHbqycqGHsislcqWGIbGyaiaawEa7caGLiWoaaeaadaabdaqaaiabdggaHjabgkHiTiabdogaJbGaay5bSlaawIa7aaaacqGGSaalcqaIXaqmaiaawUfacaGLDbaaaeaacqqGMbGzcqqGVbWBcqqGYbGCaeaadaabdaqaaiabdggaHjabgkHiTiabdogaJbGaay5bSlaawIa7aiabg6da+maaemaabaGaemyyaeMaeyOeI0IaemOyaigacaGLhWUaayjcSdGaey4jIK9aaqWaaeaacqWGHbqycqGHsislcqWGJbWyaiaawEa7caGLiWoacqGH+aGpdaabdaqaaiabdkgaIjabgkHiTiabdogaJbGaay5bSlaawIa7aaaaaiaawUhaaaaa@022C@

*value not relevant since zero saturation causes white color for any hue in this case

The calculation can also be viewed as first assigning specific hue values (0 ≤ *H*_*a *_<*H*_*b *_<*H*_*c *_< 1) to each of the three datapoints (e.g. red to *a*, green to *b*, and blue to *c*). The two most distant datapoints are then found. The distance is measured as the absolute value of their difference. A color gradient is then generated according to the identity of the two most distant datapoints (e.g. red to green color gradient if *a *and *b *are the most distant). The gradient may take two possible paths between the two characteristic hue values on the circular hue scale. The gradient path is chosen so that it does not cross the characteristic hue value of the third datapoint (the red to green gradient from the above example would run via the yellow hue to avoid the blue hue). The resulting hue value is then selected from the gradient according to the relative position of the third datapoint between the two most distant datapoints. So if *a *(red, hue value 0) and *b *(green, 1/3) are the most distant datapoints, the resulting hue value would be 0 (red), 1/3 (green), 1/6 (yellow) or 1/12 (orange) if *c *= *b*, *c *= *a*, |*a *- *b*| = 2 |*b *- *c*| or |*a *- *b*| = 4 |*b *- *c*|, respectively. If the values of the three compared datapoints are identical, the hue value is irrelevant since the saturation value is set to 0 resulting in white color as described in the next paragraph.

The saturation value of the color representing the three-way comparison is calculated using one of the following equations, according to the signals distribution:

Sresult={0forx≤Xmin1forx≥Xmaxx−XminXmax−XminforXmin<x<Xmax
 MathType@MTEF@5@5@+=feaafiart1ev1aaatCvAUfKttLearuWrP9MDH5MBPbIqV92AaeXatLxBI9gBaebbnrfifHhDYfgasaacH8akY=wiFfYdH8Gipec8Eeeu0xXdbba9frFj0=OqFfea0dXdd9vqai=hGuQ8kuc9pgc9s8qqaq=dirpe0xb9q8qiLsFr0=vr0=vr0dc8meaabaqaciaacaGaaeqabaqabeGadaaakeaacqWGtbWudaWgaaWcbaGaemOCaiNaemyzauMaem4CamNaemyDauNaemiBaWMaemiDaqhabeaakiabg2da9maaceqabaqbaeqabmWaaaqaaiabicdaWaqaaiabbAgaMjabb+gaVjabbkhaYbqaaiabdIha4jabgsMiJkabdIfaynaaBaaaleaaieGacqWFTbqBcqWFPbqAcqWFUbGBaeqaaaGcbaGaeGymaedabaGaeeOzayMaee4Ba8MaeeOCaihabaGaemiEaGNaeyyzImRaemiwaG1aaSbaaSqaaiab=1gaTjab=fgaHjab=Hha4bqabaaakeaadaWcaaqaaiabdIha4jabgkHiTiabdIfaynaaBaaaleaacqWFTbqBcqWFPbqAcqWFUbGBaeqaaaGcbaGaemiwaG1aaSbaaSqaaiab=1gaTjab=fgaHjab=Hha4bqabaGccqGHsislcqWGybawdaWgaaWcbaGae8xBa0Mae8xAaKMae8NBa4gabeaaaaaakeaacqqGMbGzcqqGVbWBcqqGYbGCaeaacqWGybawdaWgaaWcbaGae8xBa0Mae8xAaKMae8NBa4gabeaakiabgYda8iabdIha4jabgYda8iabdIfaynaaBaaaleaacqWFTbqBcqWFHbqycqWF4baEaeqaaaaaaOGaay5Eaaaaaa@7B16@

where *x *corresponds to the distance between the two most distant signal intensities and *X*_*min *_and *X*_*max *_correspond to the beginning and the end of a scale of interest (0 ≤ *X*_*min *_<*X*_*max*_). The color saturation then indicates the extent of the three-way difference between the compared signal intensities. This procedure provides what we coin as an absolute three-way difference. If the distance between the two most distant corresponding datapoints (*x*) in the formula above is divided by Max [|*a*|, |*b*|, |*c*|, *x*], we coin this result as a relative three-way difference. Multiplying the corresponding saturation values from the absolute and relative three-way comparison results amplifies differences significant in both absolute and relative terms (absolute × relative three-way difference). The resulting saturation values from any of the results can further be modified to suppress/enhance big/small values (by raising them to a certain power for example).

The brightness value of the color representing the three-way comparison is set to 1 by default. However, the brightness can be used to encode additional information relating to the three-way comparison. One possibility is to use the brightness to extend the scale representing the extent of three-way difference. Once saturation reaches the maximum along the scale axis, the brightness could be lowered to a certain degree, causing darkening of the color. The color gradients along the signal intensity scale could thus be further extended. Another possibility is to use the brightness value to overlay additional data (e.g. one of the three compared datasets) onto the three-way comparison result (Figure 3b).

The scale of interest, along which three values are compared, is not always linear. For example, the most different value among three values is not necessarily the one whose distance (absolute value of the difference) from the others is greatest, on a linear scale. In such cases, it is essential to preprocess the compared values accordingly (e.g. by taking the logarithm of the three values) prior to the calculation of a color representing the three-way comparison.

### Three-way comparisons of metabolite profiles

A Mathematica (Wolfram Research, Inc.) package TriDAMP was implemented to facilitate direct three-way comparisons of raw metabolite profiles. This package is an extension for MathDAMP [3] and is available for academic use upon request to the authors. In addition to the generation of the three-way comparison visualizations, the TriDAMP package further facilitates filtering of the results according either to the extent of the difference, to the distribution of the three compared datapoints (which of them must or must not differ), or to statistical significance when comparing three groups of replicates. Overlaid extracted ion chromatograms from the compared normalized datasets corresponding to the vicinities of the most significant three-way differences can be generated in a ranked order. User modifications of the TriDAMP code could make it applicable to data other than that resulting from metabolomics analysis. More complete information about the TriDAMP package is available by referring to the online documentation [14].

## List of abbreviations

CE – capillary electrophoresis

TOFMS – time-of-flight mass spectrometry

DEM – diethylmaleate

BSO – buthionine sulfoximine

## Authors' contributions

RB conceived the color-coding approach and implemented the TriDAMP package for direct three-way comparisons of raw metabolite profiles. All co-authors supported the evaluation of the color-coding approach and the TriDAMP package. The manuscript and the online documentation were written by RB and MR with inputs from all co-authors.

## References

[B1] Eisen MB, Spellman PT, Brown PO, Botstein D (1998). Cluster analysis and display of genome-wide expression patterns. Proc Natl Acad Sci USA.

[B2] Soga T, Baran R, Suematsu M, Ueno Y, Ikeda S, Sakurakawa T, Kakazu Y, Ishikawa T, Robert M, Nishioka T, Tomita M (2006). Differential metabolomics reveals ophthalmic acid as an oxidative stress biomarker indicating hepatic glutathione consumption. J Biol Chem.

[B3] Baran R, Kochi H, Saito N, Suematsu M, Soga T, Nishioka T, Robert M, Tomita M (2006). MathDAMP: a package for differential analysis of metabolite profiles. BMC Bioinformatics.

[B4] Hedenfalk I, Duggan D, Chen Y, Radmacher M, Bittner M, Simon R, Meltzer P, Gusterson B, Esteller M, Kallioniemi OP, Wilfond B, Borg A, Trent J (2001). Gene-expression profiles in hereditary breast cancer. N Engl J Med.

[B5] Kyng KJ, May A, Kolvraa S, Bohr VA (2003). Gene expression profiling in Werner syndrome closely resembles that of normal aging. Proc Natl Acad Sci USA.

[B6] Mueller A, O'Rourke J, Chu P, Kim CC, Sutton P, Lee A, Falkow S (2003). Protective immunity against Helicobacter is characterized by a unique transcriptional signature. Proc Natl Acad Sci USA.

[B7] Laun P, Ramachandran L, Jarolim S, Herker E, Liang P, Wang J, Weinberger M, Burhans DT, Suter B, Madeo F, Burhans WC, Breitenbach M (2005). A comparison of the aging and apoptotic transcriptome of Saccharomyces cerevisiae. FEMS Yeast Res.

[B8] Forner F, Foster LJ, Campanaro S, Valle G, Mann M (2006). Quantitative proteomic comparison of rat mitochondria from muscle, heart, and liver. Mol Cell Proteomics.

[B9] Smith AR (1978). Color gamut transform pairs. ACM SIGGRAPH Computer Graphics.

[B10] Wyrick JJ, Holstege FC, Jennings EG, Causton HC, Shore D, Grunstein M, Lander ES, Young RA (1999). Chromosomal landscape of nucleosome-dependent gene expression and silencing in yeast. Nature.

[B11] Simon I, Barnett J, Hannett N, Harbison CT, Rinaldi NJ, Volkert TL, Wyrick JJ, Zeitlinger J, Gifford DK, Jaakkola TS, Young RA (2001). Serial regulation of transcriptional regulators in the yeast cell cycle. Cell.

[B12] DeRisi JL, Iyer VR, Brown PO (1997). Exploring the metabolic and genetic control of gene expression on a genomic scale. Science.

[B13] Arakawa K, Kono N, Yamada Y, Mori H, Tomita M (2005). KEGG-based pathway visualization tool for complex omics data. In Silico Biol.

[B14] TriDAMP. http://mathdamp.iab.keio.ac.jp/tridamp/.

